# Supplementary material for: DNA-PAINT MINFLUX nanoscopy
Source: Nat Methods. 2022 Sep 1;19(9):1072–5. doi: 10.1038/s41592-022-01577-1 (PMC9467913; doi:10.1038/s41592-022-01577-1)
Supplement: Supplementary file 1 — Supplementary Table 1, Notes and Figs. 1–4. [file 41592_2022_1577_MOESM1_ESM.pdf]

nature portfolio

https://doi.org/10.1038/s41592-022-01577-1

Supplementary information

DNA-PAINT MINFLUX nanoscopy

In the format provided by the authors and unedited

DNA-PAINT MINFLUX Nanoscopy

Lynn M. Ostersehlt, Daniel C. Jans, Anna Wittek, Jan Keller-Findeisen, Kaushik Inamdar, Steffen J. Sahl, Stefan W. Hell, and Stefan Jakobs

Supplementary Table 1
Supplementary Notes 2
Performance indicators of DNA-PAINT MINFLUX recordings 2
Influence of the laser power on DNA-PAINT MINFLUX performance 4
Influence of the detection pinhole size on DNA-PAINT MINFLUX performance 6
Influence of imager concentration on DNA-PAINT MINFLUX performance 8
Optimal parameter selection in DNA-PAINT MINFLUX 9
Possible further improvements 10
References 11

Supplementary Table 1. Imager concentrations used and localization precisions (σ_r, σ_z) of the localizations represented in Figure 1 and Figure 2. Localization precisions achievable by combining all localizations of the same event (σ_{rc}, σ_{zc}) (see: Methods/ MINFLUX data analysis/ Quantification) and FRC_{xy} resolution of the images in xy (see: Methods/ MINFLUX data analysis/ FRC calculations). Note that the FRC resolution is strongly influenced by sample parameters such as labeling density or existing feature sizes and does not reflect the optical resolving power of the instrument alone.

Figure	Imager concentration	Localization precision σ_r (σ_z)	Combined localization precision σ_{rc} (σ_{zc})	FRC_{xy} resolution
Figure 1a	2 nM	2.7 nm	1.1 nm	9.2 nm
Figure 1b	2.5 nM	2.6 nm	0.8 nm	4.7 nm
Figure 1c	0.5 nM	2.3 nm	1.1 nm	7.8 nm
Figure 1d	2 nM	2.4 nm	0.8 nm	6.0 nm
Figure 1e	n. a.	3.0 nm	1.4 nm	9.0 nm
Figure 1f	2 nM	2.7 nm	0.9 nm	14.9 nm
Figure 2 TOM70	2 nM	5.6 nm (3.1 nm)	2.4 nm (0.8 nm)	17.8 nm
Figure 2 Mic60	1 nM	5.4 nm (3.1 nm)	2.0 nm (0.8 nm)	32.1 nm
Figure 2 ATP5B	1 nM	5.1 nm (3.1 nm)	1.9 nm (0.8 nm)	55.5 nm

Supplementary Notes

Performance indicators of DNA-PAINT MINFLUX recordings

We systematically explored the influence of the experimental key variables (excitation laser power, pinhole size and imager concentration) on DNA-PAINT MINFLUX recordings. Specifically, the influence on the time between valid events (t_{btw}), the background emission frequency (f_{bg}), the center-frequency-ratio (CFR) and the localization precision (σ_r) were determined, as together these four parameters provide a measure of the image quality, the average success of the localization processes, and the time for recording a MINFLUX image. These parameters are calculated according to their definition given in Methods, MINFLUX 2D Analysis.

The idle time between two valid molecule binding events (t_{btw}) is a major determinant of the overall recording speed in MINFLUX nanoscopy, as the molecules are recorded sequentially.

The background emission frequency (f_{bg}) is continuously estimated by the MINFLUX microscope in between valid events and is used by the system to identify emission events and to correct emission frequencies of localization events.

The center-frequency-ratio (CFR*)* is a parameter calculated during image acquisition by the MINFLUX software and is used as an internal abort criterion in the first and the third 2D MINFLUX iteration steps at each localization attempt. The CFR is defined as the ratio between the effective, background corrected emission frequency determined at the central position of the MINFLUX targeted coordinate pattern (TCP) over the average effective emission frequency at the outer positions. The CFR is small when the central position of the probing donut is placed on the molecule and the CFR increases when the central position of the donut in the MINFLUX targeted coordinate pattern (TCP) deviates from the molecule position. Its value is also influenced by the effectiveness of the background correction.

Because the CFR is only a general indicator for the localization quality, we also directly determined the localization precision in the measurements. To estimate the average localization precision within one measurement, we choose the median of σ_r of all events with ≥4 localizations.

To systematically characterize the influence of varying excitation laser powers, pinhole sizes and imager concentrations on t_{btw}, f_{bg}, CFR and σ_r we recorded DNA-PAINT MINFLUX images of a well-established intracellular model structure, namely nuclear pores in cultivated human cells. To this end, genome edited HeLa cells expressing mEGFP-Nup107 were chemically fixed and labeled with anti-GFP nanobodies that were coupled to a docking DNA-

oligo. The complementary DNA-oligo coupled to Atto655 was used as an imager. Within one quantification measurement series (see also Methods, MINFLUX measurements), all experimental variables but one were kept constant. All measurements within a series were repeated three times on different days.

Influence of the laser power on DNA-PAINT MINFLUX performance

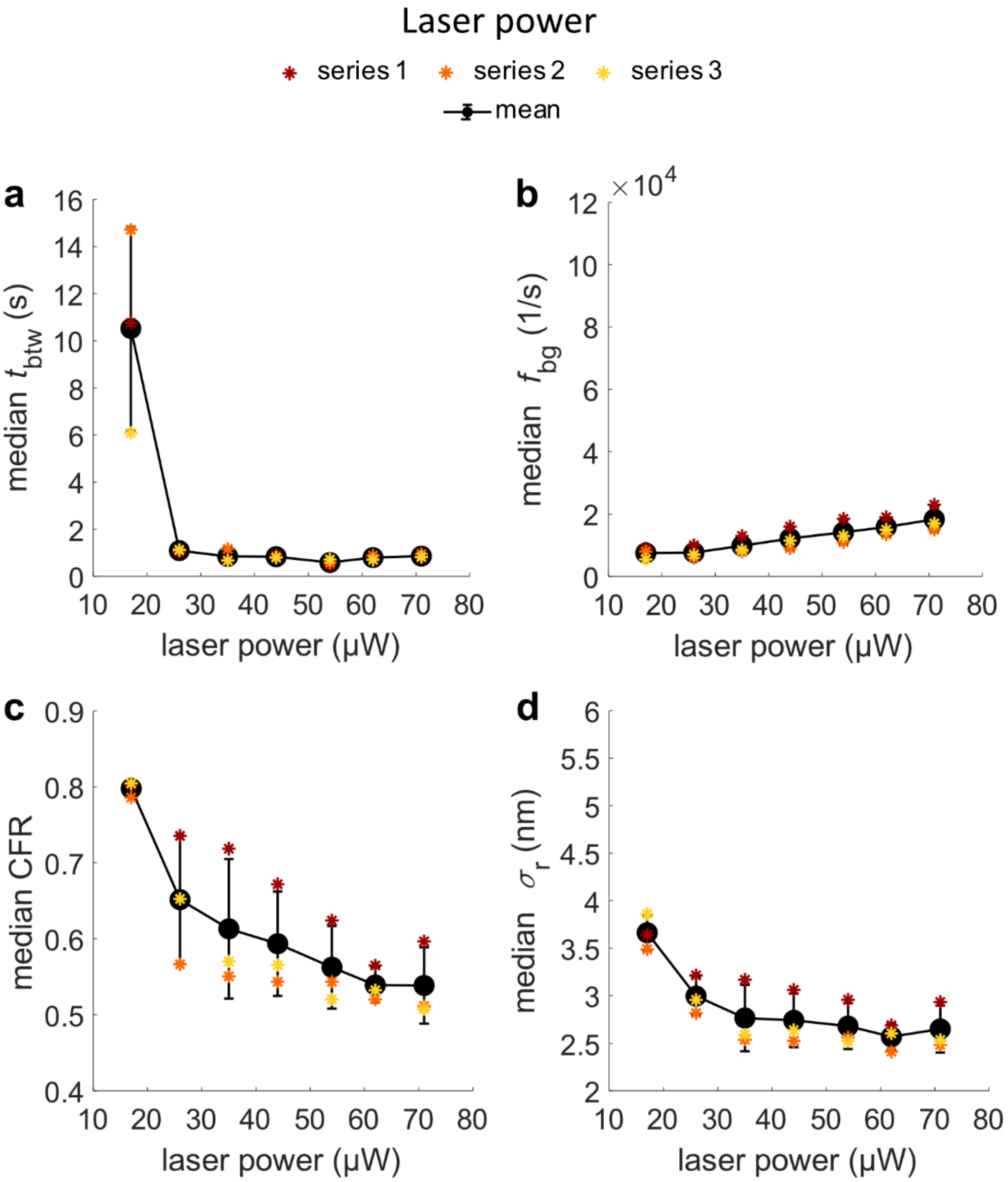

Supplementary Figure 1. Influence of the laser power on the parameters t_{btw} , f_{bg}, CFR and σ_r. Nup107-mEGFP cells were fixed and labelled with an anti-GFP nanobody coupled to a docking strand. 1 μm^2 ROIs were imaged for an hour each, using a pinhole size of 0.45 AU and an imager concentration of 2 nM. The laser powers given refer to the power in the sample at the first iteration of the MINFLUX sequence. At the last iteration of the sequence, the power is six times higher. Colored asterisks represent the median of the respective parameter within one measurement series. Black dots represent the mean of the three biologically independent measurement series and error bars represent the standard deviation from the mean.

In the MINFLUX sequence used, the laser power is increased six fold from the first to the final iteration. Consequently, the initial laser power could be maximally set to 71 µW (in the sample; 16% of the available laser power). To characterize the influence of the laser power on t_{btw}, f_{bg}, CFR and σ_r we varied the laser power between 17 and 71 µW in the first iteration (4 % - 16 %), which also corresponds to a variation of the laser power in all other iterations. In this measurement series, all images were taken with an imager concentration of 2 nM and a pinhole size of 0.45 Airy units (AU).

At laser powers below 26 µW in the first iteration (6 %), the t_{btw} increased, presumably, because at too low laser intensities the likelihood of events with sufficient detected photons to cross the photon thresholds in the MINFLUX iteration scheme decreases (Supplementary Fig. 1a). Above a minimal laser power threshold, the t_{btw} was largely independent of the laser power. This can be attributed to the fact that in DNA-PAINT the single-molecule event kinetics are primarily determined by the binding kinetics of the imager to the docking strand, and not by activation light, as in previous MINFLUX implementations.

Higher laser powers led to an increased f_{bg} (Supplementary Fig. 1b). This can be explained by a stronger excitation of free imager in the sample.

With increasing laser power, the experimentally observed median CFR decreased (Supplementary Note Fig. 1c). For a background-free DNA-PAINT MINFLUX measurement, we would expect the CFR to be independent of the laser power. However, when imaging a real biological sample, background is inevitable. In DNA-PAINT background is especially high due to the free imager in the sample. The MINFLUX software applies an automated adaptive background correction on the estimation of the CFR. As we observe a decrease of the CFR with increasing laser power, we assume that the algorithm does not completely correct for the background.

Similar to the CFR, also the median of σ_r slightly decreased with increasing laser power (Supplementary Fig. 1d). This is likely a side effect of the finite dwell time per targeted coordinate, which at higher laser powers results in a slightly higher number of collected photons above the threshold that must be reached for the localization to be accepted.

Influence of the detection pinhole size on DNA-PAINT MINFLUX performance

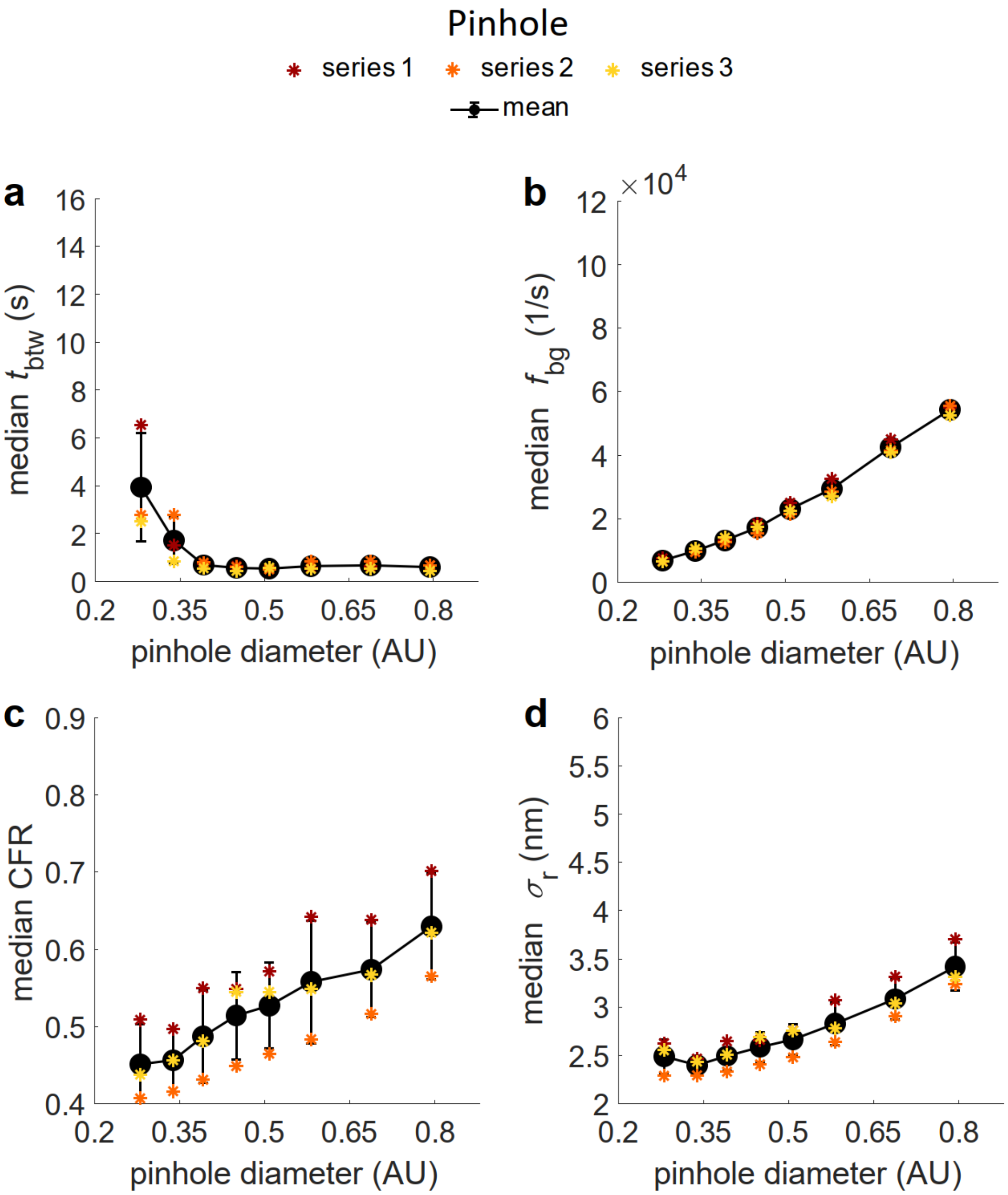

Supplementary Figure 2. Influence of the pinhole size on the parameters t_{btw}, f_{bg}, CFR and σ_r. Nup107-mEGFP cells were fixed and labelled with an anti-GFP nanobody coupled to a docking strand. 1 μm^2 ROIs were imaged for an hour each, using a laser power of 71 µW in the sample in the first iteration and an imager concentration of 2 nM. Colored asterisks represent the median of the respective parameter within one measurement series. AU: Airy units. Black dots represent the mean of the three biologically independent measurement series and error bars represent the standard deviation from the mean.

We analysed the influence of different pinhole sizes on t_{btw}, f_{bg}, CFR and σ_r. For this, we chose to vary the size of the pinhole in a range of 0.28 - 0.79 AU. The images were recorded with a laser power of 71 µW in the first iteration and 2 nM imager concentration.

Above a threshold (~ 0.4 AU), we found t_{btw} to reach a constant plateau (Supplementary Fig. 2a). The increase of t_{btw} at small pinhole sizes is expected, as when decreasing the pinhole size, not only background photons are rejected, but also photons emitted by the localized molecule. Consequently, less and less signal is detected until an increasing number of localization attempts no longer passes the photon thresholds of the MINFLUX iteration sequence.

The f_{bg} increased with larger pinhole sizes (Supplementary Fig. 2b). This is immediately explained by increased photon counts from the free imager in the buffer.

The CFR increased almost linearly with the pinhole size (Supplementary Fig. 2c). Calculations that take into account an increasing background related to the pinhole size but do not consider an adaptive background correction, also suggest an approximately linear relationship between pinhole size and CFR (Supplementary Fig. 3), similar to the measured data. Again, this observation suggests that the background subtraction performed by the MINFLUX software does not fully compensate for the background when using DNA-PAINT. With smaller pinhole sizes, the experimentally determined localization precision improved (Supplementary Fig. 2d) down to a pinhole size of 0.34 AU. At even smaller pinhole sizes, presumably too few photons were detected to improve σ_r further.

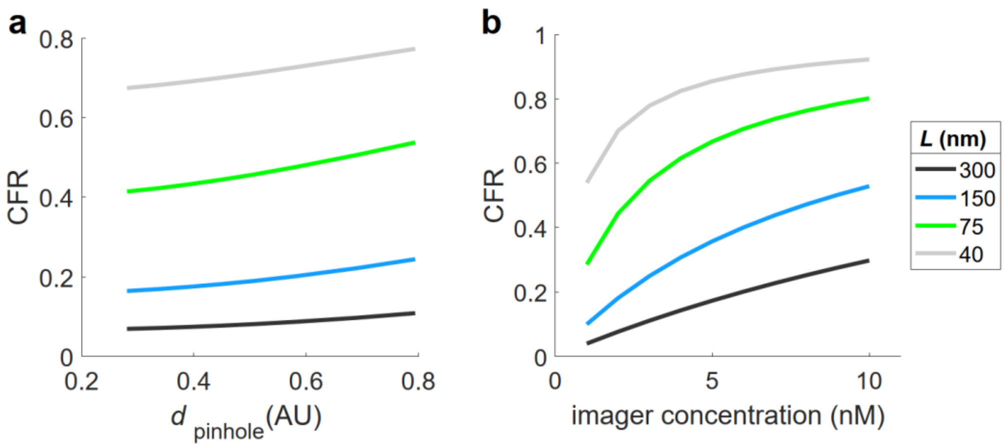

Supplementary Figure 3. CFR simulations for varying pinhole diameter at 2 nM imager concentration (a) and varying imager concentration at a pinhole size of 0.45 AU (b). The CFR was calculated in both cases as described in (Methods, CFR Calculations) for one MINFLUX iteration using a targeted coordinate pattern (TCP) with one central exposure and six outer exposures arranged on a circle with diameter L.

Influence of imager concentration on DNA-PAINT MINFLUX performance

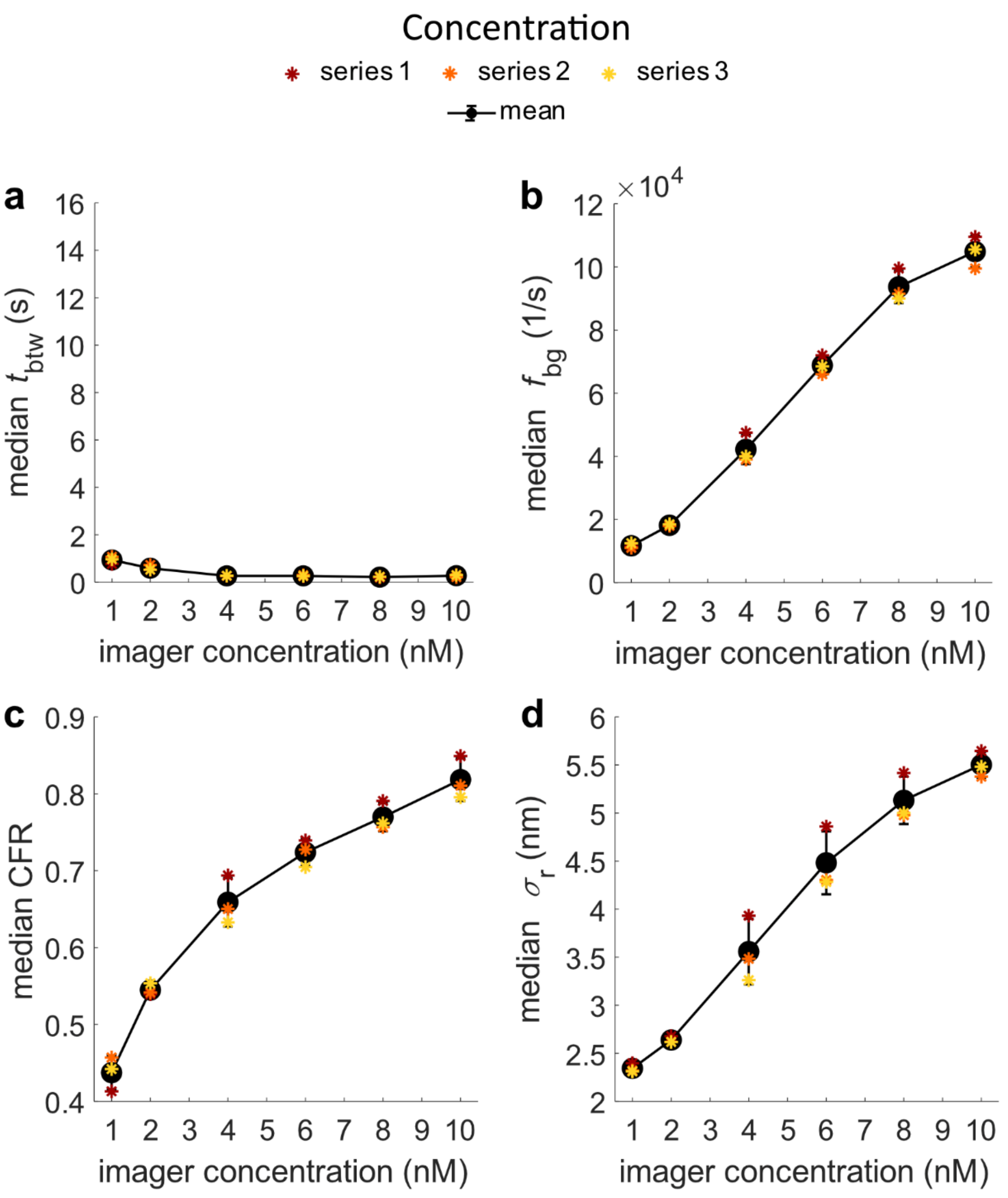

Supplementary Figure 4. Influence of the imager concentration on the parameters t_{btw}, f_{bg}, CFR and σ_r. Nup107-mEGFP cells were fixed and labelled with an anti-GFP nanobody coupled to a docking strand. 1 µm^2 ROIs were imaged for an hour each, using a pinhole size of 0.45 AU and a laser power of 71 µW in the sample in the first iteration. Colored asterisks represent the median of the respective parameter within one measurement series. Black dots represent the mean of the three biologically independent measurement series and error bars represent the standard deviation from the mean.

The influence of the imager concentration on the DNA-PAINT MINFLUX imaging parameters was analysed. The imager strand concentration was varied between 1 and 10 nM. The laser power was set to 71 µW in the first iteration, and a pinhole size of 0.45 AU was used.

At very low imager concentrations, t_{btw} increased (Supplementary Fig. 4a). This was expected, as the number of binding events scales linearly with the concentration of the imager at low concentrations. Above ~ 4 nM imager, t_{btw} reached a plateau. This demonstrates that t_{btw} is rather insensitive against the imager concentration, once the lower threshold is passed. We predict that the t_{btw} might increase again at higher imager concentrations outside of the tested concentration range, because we expect at very high imager concentrations an increasing number of aborted localization events due to multiple fluorophores binding within the examined MINFLUX localization region.

The f_{bg} increased with higher imager concentrations (Supplementary Fig. 4b). A linear dependence of background on imager concentration is to be expected. However, the shape of the curve indicates a slightly non-linear relationship, suggesting a not fully functional background detection by the microscope software in DNA-PAINT.

The CFR increased with increasing imager concentrations (Supplementary Fig. 4c). Computing this relationship without background correction, assuming a background intensity $I_{bg}(c)$ which depends linearly on the imager strand concentration and a background independent molecule intensity I_m, results in $\mathrm{CFR}(c) \sim \frac{I_{bg}\,(\mathrm{c})}{I_{bg}\,(\mathrm{c}) + I_m}$, which reflects the experimental data well for small diameter L of the MINFLUX excitation pattern (Supplementary Fig. 3).

The localization precision decreases with an increasing imager concentration (Supplementary Fig. 4d). We assume that with higher imager concentrations not only the background increases, but also the likelihood of a second imager molecule binding in spatial proximity to a localized binding event rises. These two factors will result in the decrease of the median σ_r.

Optimal parameter selection in DNA-PAINT MINFLUX

Together, these data show that in DNA-PAINT MINFLUX imaging an appropriate imager concentration is a key determinant of the localization precision. However, at too low imager concentrations t_{btw} increases strongly. The pinhole size has opposed effects on the localization precision and on t_{btw}, requiring the identification of an optimal pinhole size. The localization precision increases with higher power powers until it reaches a plateau, and at the laser

intensities available, we did not observe any decrease of t_{btw} above a threshold of 26 µW in the first iteration. The MINFLUX microscope largely behaves as expected for an imaging system with only partial background subtraction in the estimation of the CFR.

In conclusion, a good starting point for DNA-PAINT MINFLUX measurements using Atto655 is a laser power of at least 62 µW in the first iteration, a pinhole size of 0.45 AU and, for nuclear pore imaging, an imager concentration of 2 nM (the imager concentration has to be adapted to the target binding sites density).

For the use of other dyes, the imaging parameters presumably need to be adjusted. This study shows that the imager background is a major factor influencing the localization performance in DNA-PAINT MINFLUX nanoscopy. Therefore, it is advisable to start optimizing parameters with a low imager concentration (without extending the recording time to unacceptable values). A small pinhole should be chosen, and a sufficiently high laser power is required to collect enough photons during one binding event.

Possible further improvements

DNA-PAINT MINFLUX nanoscopy has distinct advantages over conventional MINFLUX nanoscopy, most notably the possibility of unlimited multiplexing and the lack of a need for dedicated buffer adjustments.

However, free imager causes an increase in the background emission frequency (f_{bg}), and the challenge of long recoding times remains. Both challenges are also known from standard DNA-PAINT nanoscopy.

Several approaches to reduce the background problem in DNA-PAINT nanoscopy have been reported. This includes the use of Förster resonance energy transfer (FRET)-based probes[1, 2], caged, photo-activatable dyes[3], as well as fluorogenic DNA-PAINT probes[4]. Presumably, these or related approaches would also benefit DNA-PAINT MINFLUX nanoscopy.

In the DNA-PAINT implementation used in this study, we relied on standard, commercially available imager strands. Thereby we localized each molecule more than 20 times on average, while the imager strand was bound to the docking strand. A probe with a moderately higher off-rate would presumably save time, as fewer localizations per event would be collected, without unacceptably deteriorating the localization precision.

Using a probe with a higher on-rate would additionally allow for lower imager concentration and thereby reduce the background, without extending the idle time between two valid molecule binding events (t_{btw}). Indeed, several studies report on the design of optimized DNA

sequences and buffer optimization in order to minimize the time between events in DNA-PAINT nanoscopy and thereby accelerate the recording[5-8]. This resulted in an up to 100-fold speed-up in imaging[7]. Other concepts to accelerate DNA-PAINT nanoscopy relied on the preloading of DNA-PAINT imager strands with Argonaute proteins[9].

In addition to speeding up the recoding by modulating the binding kinetics of the imager strand, we assume that there is potential in tailoring the MINFLUX sequence to DNA-PAINT labelling. For this study, we relied on the generic MINFLUX sequence provided by the microscope manufacturer. This has not been optimized for DNA-PAINT and we assume that further improvements in imaging time and localization quality are possible when this sequence would be specifically tailored. Concretely, the number of iterations, the photon thresholds, the number of localization attempts for one event and the sizes of the TCP diameter L in the iterations could be adapted.

Ultimately, we predict that accelerating MINFLUX nanoscopy will require parallelization of the localization process by changing the instrument design.

References

1. Auer, A., Strauss, M.T., Schlichthaerle, T. & Jungmann, R. Fast, Background-Free DNA-PAINT Imaging Using FRET-Based Probes. *Nano Lett* **17**, 6428-6434 (2017).
2. Lee, J., Park, S., Kang, W. & Hohng, S. Accelerated super-resolution imaging with FRET-PAINT. *Mol Brain* **10**, 63 (2017).
3. Jang, S., Kim, M. & Shim, S.H. Reductively Caged, Photoactivatable DNA-PAINT for High-Throughput Super-resolution Microscopy. *Angew Chem Int Ed Engl* **59**, 11758-11762 (2020).
4. Chung, K.K. et al. Fluorogenic probe for fast 3D whole-cell DNA-PAINT. *bioRxiv*, 2020.2004.2029.066886 (2020).
5. Schickinger, M., Zacharias, M. & Dietz, H. Tethered multifluorophore motion reveals equilibrium transition kinetics of single DNA double helices. *Proc Natl Acad Sci U S A* **115**, E7512-E7521 (2018).
6. Schueder, F. et al. An order of magnitude faster DNA-PAINT imaging by optimized sequence design and buffer conditions. *Nat Methods* **16**, 1101-1104 (2019).
7. Strauss, S. & Jungmann, R. Up to 100-fold speed-up and multiplexing in optimized DNA-PAINT. *Nat Methods* **17**, 789-791 (2020).
8. Clowsley, A.H. et al. Repeat DNA-PAINT suppresses background and non-specific signals in optical nanoscopy. *Nat Commun* **12**, 501 (2021).
9. Filius, M. et al. High-Speed Super-Resolution Imaging Using Protein-Assisted DNA-PAINT. *Nano Lett* **20**, 2264-2270 (2020).
