# Supplementary material for: DNA-PAINT MINFLUX nanoscopy
Source: Nat Methods. 2022 Sep 1;19(9):1072–5. doi: 10.1038/s41592-022-01577-1 (PMC9467913; doi:10.1038/s41592-022-01577-1)
Supplement: Supplementary file 2 — Reporting Summary [file 41592_2022_1577_MOESM2_ESM.pdf]

nature portfolio

Corresponding author(s): Stefan W. Hell; Stefan Jakobs

Last updated by author(s): Jun 24, 2022

Reporting Summary

Nature Portfolio wishes to improve the reproducibility of the work that we publish. This form provides structure for consistency and transparency in reporting. For further information on Nature Portfolio policies, see our Editorial Policies and the Editorial Policy Checklist.

Statistics

For all statistical analyses, confirm that the following items are present in the figure legend, table legend, main text, or Methods section.

n/a	Confirmed	
☐	☒	The exact sample size (*n*) for each experimental group/condition, given as a discrete number and unit of measurement
☐	☒	A statement on whether measurements were taken from distinct samples or whether the same sample was measured repeatedly
☒	☐	The statistical test(s) used AND whether they are one- or two-sided<br>*Only common tests should be described solely by name; describe more complex techniques in the Methods section.*
☒	☐	A description of all covariates tested
☒	☐	A description of any assumptions or corrections, such as tests of normality and adjustment for multiple comparisons
☐	☒	A full description of the statistical parameters including central tendency (e.g. means) or other basic estimates (e.g. regression coefficient) AND variation (e.g. standard deviation) or associated estimates of uncertainty (e.g. confidence intervals)
☒	☐	For null hypothesis testing, the test statistic (e.g. *F*, *t*, *r*) with confidence intervals, effect sizes, degrees of freedom and *P* value noted<br>*Give P values as exact values whenever suitable.*
☒	☐	For Bayesian analysis, information on the choice of priors and Markov chain Monte Carlo settings
☒	☐	For hierarchical and complex designs, identification of the appropriate level for tests and full reporting of outcomes
☒	☐	Estimates of effect sizes (e.g. Cohen's *d*, Pearson's *r*), indicating how they were calculated

Our web collection on statistics for biologists contains articles on many of the points above.

Software and code

Policy information about availability of computer code

Data collection	Imspector (version 16.3.11647M-devel-win64-MINFLUX, Abberior Instruments)
Data analysis	Imspector (version 16.3.11647M-devel-win64-MINFLUX, Abberior Instruments), MATLAB R2018b, https://doi.org/10.5281/zenodo.6563100

For manuscripts utilizing custom algorithms or software that are central to the research but not yet described in published literature, software must be made available to editors and reviewers. We strongly encourage code deposition in a community repository (e.g. GitHub). See the Nature Portfolio guidelines for submitting code & software for further information.

Data

Policy information about availability of data

All manuscripts must include a data availability statement. This statement should provide the following information, where applicable:

- Accession codes, unique identifiers, or web links for publicly available datasets
- A description of any restrictions on data availability
- For clinical datasets or third party data, please ensure that the statement adheres to our policy

All DNA-PAINT MINFLUX localization data have been deposited at https://doi.org/10.5281/zenodo.6563100. The raw data as provided by the microscope software are available at https://doi.org/10.5281/zenodo.6562764

Field-specific reporting

Please select the one below that is the best fit for your research. If you are not sure, read the appropriate sections before making your selection.

☒ Life sciences ☐ Behavioural & social sciences ☐ Ecological, evolutionary & environmental sciences

For a reference copy of the document with all sections, see nature.com/documents/nr-reporting-summary-flat.pdf

Life sciences study design

All studies must disclose on these points even when the disclosure is negative.

Sample size	No sample-size calculation was performed. The manuscript reports the demonstration of an imaging method, but draws no biological conclusions, and does not examine or compare different biological conditions. This is not a life science study with coparative analyes of a certain sample size
Data exclusions	No data was excluded from the analysis
Replication	All attempts of replication were successful. All experiments were repeated three or more times with similar results.
Randomization	No randomization was performed. This is not a life science study with comparative analyses of biological situations.
Blinding	No blinding was performed. There is no comparison of different biological situations performed in this work.

Reporting for specific materials, systems and methods

We require information from authors about some types of materials, experimental systems and methods used in many studies. Here, indicate whether each material, system or method listed is relevant to your study. If you are not sure if a list item applies to your research, read the appropriate section before selecting a response.

Materials & experimental systems

n/a	Involved in the study	
☐	☒	Antibodies
☐	☒	Eukaryotic cell lines
☒	☐	Palaeontology and archaeology
☒	☐	Animals and other organisms
☒	☐	Human research participants
☒	☐	Clinical data
☒	☐	Dual use research of concern

Methods

n/a	Involved in the study	
☒	☐	ChIP-seq
☒	☐	Flow cytometry
☒	☐	MRI-based neuroimaging

Antibodies

Antibodies used	anti-Mitofilin (IMMT/Mic60) (10179-1-AP, Proteintech)<br>anti-ATP Synthase Subunit beta ATPB [4.3E8.D10] (ab5432, Abcam)<br>MASSIVE-TAG-Q anti-GFP nanobody (Massive Photonics; no catalogue number available)<br>FluoTag-Q anti-GFP anti-GFP single domain antibody (conjugated with AlexaFluor647) (N0301-AF647-L, NanoTag Biotechnologies)<br><br>IgG anti-rabbit (MASSIVE-AB 1-PLEX, Massive Photonics)<br>IgG anti-mouse (MASSIVE-AB 1-PLEX, Massive Photonics)
Validation	anti-Mitofilin (IMMT/Mic60) (10179-1-AP, Proteintech) - we demonstrated the specificity of this antibody with Mitofilin/Mic60 KO cells in a previous publication (Stephan et al, EMBOJ, 2020). anti-ATP Synthase Subunit beta ATPB [4.3E8.D10] (ab5432, Abcam) - this antibody has been tested and used for different applications in various publications (e.g. Steinberg et al, Nat Commun, 2020; Diokmetzidou, J Cell Sci, 2016; Jans et al, PNAS, 2013; etc.), MASSIVE-TAG-Q anti-GFP nanobody (Massive Photonics) and FluoTag-Q anti-GFP single domain antibody (NanoTag Biotechnologies) - both are the same single domain antibodies differently conjugated. This single domain antibody has been tested and used for different applications in various publications (e.g. Sograte-Idrissi et al, Cells, 2019; Oleksiievets et al., Commun Biol, 2022; Seitz et al, Sci Rep, 2019; Thevathasa et al, Nat Methods, 2019)

Eukaryotic cell lines

Policy information about cell lines

Cell line source(s)	CLS Cell Lines Services GmbH, Eppelheim, Germany (NUP96-mEGFP cell line U2OS-CRISPR-NUP96-mEGFP clone #195

Cell line source(s)	(300174)21 and NUP107-mEGFP cell line HK-2xZFN-mEGFP-Nup107). Cell lines HMGA1-rsEGFP2, Zyxin-rsEGFP2, Vimentin-rsEGFP2 and TOMM70A-Dreiklang were produced from U2OS cells (American Type Culture Collection, Manassas, VA, USA) as described in (Ratz et al, Sci Rep, 2015).
Authentication	Authentification by microscopy.
Mycoplasma contamination	The cell line was tested for mycoplasma contamination and negative results were obtained.
Commonly misidentified lines (See ICLAC register)	None.
