# Supplementary material for: DNA-PAINT MINFLUX nanoscopy
Source: Nat Methods. 2022 Sep 1;19(9):1072–5. doi: 10.1038/s41592-022-01577-1 (PMC9467913; doi:10.1038/s41592-022-01577-1)
Supplement: Supplementary file 4 — MINFLUX sequences that control parameters of the MINFLUX measurement (seqIIF.json and seqDefaultIIF3d.json). [file 41592_2022_1577_MOESM4_ESM.zip › Supplement_17_3_2022.docx]

**DNA-PAINT MINFLUX Nanoscopy**

Lynn M. Ostersehlt, Daniel C. Jans, Anna Wittek, Jan Keller-Findeisen, Steffen J. Sahl,

Stefan W. Hell, and Stefan Jakobs

[**Supplementary Methods** 1](#_Toc97142441)

[Cell Lines 1](#_Toc97142442)

[Cell culture 1](#_Toc97142443)

[Sample preparation 1](#_Toc97142444)

[Sample mounting and imaging buffer 2](#_Toc97142445)

[MINFLUX measurements 2](#_Toc97142446)

[Daily alignment of the MINFLUX nanoscope 3](#_Toc97142447)

[MINFLUX data analysis 3](#_Toc97142448)

[Image rendering 2D 6](#_Toc97142449)

[Image rendering 3D 6](#_Toc97142450)

[MINFLUX sequences 7](#_Toc97142451)

[**Supplementary Table** 8](#_Toc97142452)

[**Supplementary Figure 1** 9](#_Toc97142453)

[**Supplementary Figure 2** 10](#_Toc97142454)

[**Supplementary Figure 3** 11](#_Toc97142455)

[**Supplementary notes** 12](#_Toc97142456)

[Performance indicators of DNA-PAINT MINFLUX recordings 12](#_Toc97142457)

[Influence of the laser power on MINFLUX performance 14](#_Toc97142458)

[Influence of the detection pinhole size on MINFLUX performance 16](#_Toc97142459)

[Influence of imager concentration on MINFLUX performance 18](#_Toc97142460)

[Optimal parameter selection in DNA-PAINT MINFLUX 19](#_Toc97142461)

[Possible further improvements 20](#_Toc97142462)

[**References** 22](#_Toc97142463)

**Supplementary Methods**

**Cell Lines.** The genome edited U2OS cell lines HMGA1-rsEGFP2 (homozygous), Zyxin-rsEGFP2 (homozygous) and Vimentin-rsEGFP2 (heterozygous) were described in (1). The heterozygous TOMM70A-Dreiklang U2OS cell line was generated as described in (1). The homozygous NUP96-mEGFP cell line U2OS-CRISPR-NUP96-mEGFP clone #195 (300174) (2) and the NUP107-mEGFP cell line HK-2xZFN-mEGFP-Nup107 (300676) (3) were purchased from CLS GmbH (CLS Cell Lines Service GmbH, Eppelheim, Germany).

**Cell culture.** U2OS cells were cultivated in McCoy’s 5a medium (Thermo Fisher Scientific, Waltham, MA, USA), supplemented with 100 U/ml penicillin, 100 μg/ml streptomycin, 1 mM Na-pyruvate, and 10 % (v/v) FCS (Invitrogen, Waltham, MA, USA) at 37 °C, 5 % CO_2_. HeLa Kyoto cells were cultivated in DMEM, high glucose, GlutaMAX™ Supplement, pyruvate (Thermo Fisher Scientific, Waltham, MA, USA), supplemented with 100 U/ml penicillin, 100 μg/ml streptomycin and 10 % (v/v) FCS (Invitrogen, Waltham, MA, USA) at 37 °C, 5 % CO_2_.

**Sample preparation.** The cells were cultured for 1 day on coverslips (Marienfeld, Lauda-Königshofen, Germany) or in 8 well chambered cover slips (ibidi, Gräfelfing, Germany) and fixed in pre-warmed 8 % formaldehyde in PBS for 10 minutes. Fixed cells were permeabilized with 0.5 % (v/v) Triton X-100 in PBS for 5 min. NUP107-mEGFP cells were fixed in 2.4 % formaldehyde in PBS for 30 min at room temperature and after fixation incubated with 0.1 M NH_4_Cl in PBS for 5 min. Then, NUP107-mEGFP cells were permeabilized with 0.25 % (v/v) Triton X-100. Afterwards, all cells were blocked in antibody incubation buffer (Massive Photonics, Gräfelfing, Germany) for ~30 min. The cells were incubated for 1 h with the MASSIVE-TAG-Q anti-GFP single domain antibody (Massive Photonics) in antibody incubation buffer (Massive Photonics) at a dilution of 1:100. The cells were then washed three times with 1x washing buffer (Massive Photonics). For multiplexing, the cells were fixed, permeabilized and blocked as described above. Afterwards the cells were incubated for 1 h at room temperature with primary antibodies against Mic60 (Proteintech) at a concentration of 1.235 µg/ml and ATP synthase subunit beta (Abcam) at a concentration of 5 µg/ml in antibody incubation buffer (Massive Photonics). After three washing steps with PBS, the cells were incubated with polyclonal secondary antibodies coupled to DNA-PAINT docking sites, targeting mouse and rabbit IgGs (Massive Photonics) at a dilution of 1:400 each or with MASSIVE-TAG-Q anti-GFP single domain antibody (Massive Photonics) at a dilution of 1:100. The cells were then washed three times with 1x washing buffer (Massive Photonics).

**Sample mounting and imaging buffer.** For the stabilization of the samples during MINFLUX imaging, the samples were incubated with 100 µl of gold nanorods dispersion (A12-40-980-CTAB-DIH-1-25, Nanopartz Inc., Loveland, CO, USA) for 7 min, as described before (4, 5). To remove unbound nanorods, the samples were rinsed with PBS several times. For single-color DNA-PAINT imaging, aliquots (5 µM) of the DNA-PAINT imager conjugated to Atto655 (Massive Photonics) were diluted in imaging buffer (Massive Photonics) (final concentrations indicated in Supplementary Table 1). Coverslips were sealed with picodent twinsil (picodent, Wipperfürth, Germany) on cavity slides (Brand GmbH & CO KG, Wertheim, Germany). For multiplexing, 8 well chambered cover slips (ibidi) were used. After incubation with gold nanorod dispersion and washing as described above, aliquots (5 µM) of the DNA-PAINT imager (conjugated to Atto655) (Massive Photonics) transiently binding to MASSIVE-TAG-Q anti-GFP single domain antibody were diluted in imaging buffer (final concentration: 2 nM) (Massive Photonics) and added to the cells. After DNA-PAINT MINFLUX imaging, the cells were washed on the microscope stage five times with PBS and one time with imaging buffer (Massive Photonics). Subsequently, DNA-PAINT imager (conjugated to Atto655) (Massive Photonics) transiently binding to the anti-rabbit IgG was diluted (final concentration: 1 nM) and added. After recording of the second DNA-PAINT MINFLUX dataset this process was repeated and imager transiently binding to the anti-mouse IgG (final concentration: 1nM) was added.

**MINFLUX measurements.** The data were acquired on an Abberior MINFLUX microscope (Abberior Instruments, Göttingen, Germany) (5) using the Imspector software (version 16.3.11647M-devel-win64-MINFLUX, Abberior Instruments). For MINFLUX measurements, the Imspector MINFLUX sequence templates seqIIF (2D) and DefaultIIF3D (3D) provided and optimized by the manufacturer for samples with the dye Alexa Fluor 647 were used (see *MINFLUX sequences*).

Cells were identified and placed in the focus using the 488 nm confocal scan of the microscope. If necessary, the persistent binding-unbinding activity of imager strands was verified in the 642 nm confocal scan. Before starting a MINFLUX measurement, the stabilisation system of the microscope was activated. Measurements were conducted with a stabilization precision of typically below 1 nm. A region of interest was selected in the confocal scan image and laser power and pinhole size were adjusted in the software (indicated pinhole sizes in AU refer to the emission maximum of Atto655 at 680 nm). Finally, the MINFLUX measurement was started in the region of interest.

*Quantification measurement series.* In a measurement series (see also Supplementary notes) one of the experimental parameters, namely laser power, pinhole size or imager concentration, was varied, while the other parameters were kept constant. Within one measurement series, we recorded 2D MINFLUX images of labelled nuclear pores close to the cover slip and kept the image size and the recording time (1 h) constant. All images were taken with the same MINFLUX iteration sequence. Multiple regions (1 µm × 1 µm) of the lower envelope of one nucleus were measured. Each region was imaged with a different experimental parameter. Each measurement series was repeated three times on different days with fresh samples.

**Daily alignment of the MINFLUX nanoscope.** The shape of the excitation point spread function was evaluated using immobilized fluorescent beads (GATTA-BEAD R, Gattaquant GmbH, Gräfelfing, Germany) and if necessary optimized by changing the SLM (spatial light modulator) parameters. Additionally, the position of the pinhole was adjusted so that the confocal detection matched the excitation volume. If during measurement series more than one pinhole size was used, all pinhole positions were determined before starting the measurement series. The pinhole position was then adjusted prior to each measurement.

**MINFLUX data analysis.** *Data export.* Each MINFLUX measurement was exported with the Imspector software (Abberior Instruments). The exported files contained a collection of recorded parameters for all valid localizations and also included discarded non-valid localization attempts. Additional information of the measurement (laser power, etc.) was stored manually. Both were imported in a custom analysis script written in Matlab (R2018b) to calculate the following quantification parameters in an automated manner.

*Quantification.* For all calculations, only data of the last MINFLUX iterations (in 2D: 4^th^, in 3D: 9^th^, after one pre-localization iteration), which were also identified as valid (exported parameter: VLD = 1), were used.

The first quantification parameter to be calculated was the time that passes between the localization of two valid events, in short, the time between events, or $t_{\text{btw}}$. An emitting molecule is usually localized by the microscope several times in direct succession by repeating the last two MINFLUX iterations. These successive localizations are assigned to the same event via the same trace ID (exported parameter: TID). Moreover, for each individual localization the time at which its localization process started was saved (exported parameter: TIM). This allowed the determination of the start and end time of each molecule binding event. The time of the first non-valid localization attempt following a series of valid localization attempts was defined as the end time of the molecule binding event. Finally, $t_{\text{btw}}$ was calculated as the time difference between two consecutive valid events, by subtracting the end time of the first molecule from the start time of the second molecule. For each measurement the median of the first 100 events was determined as$t_{\text{btw}}$.


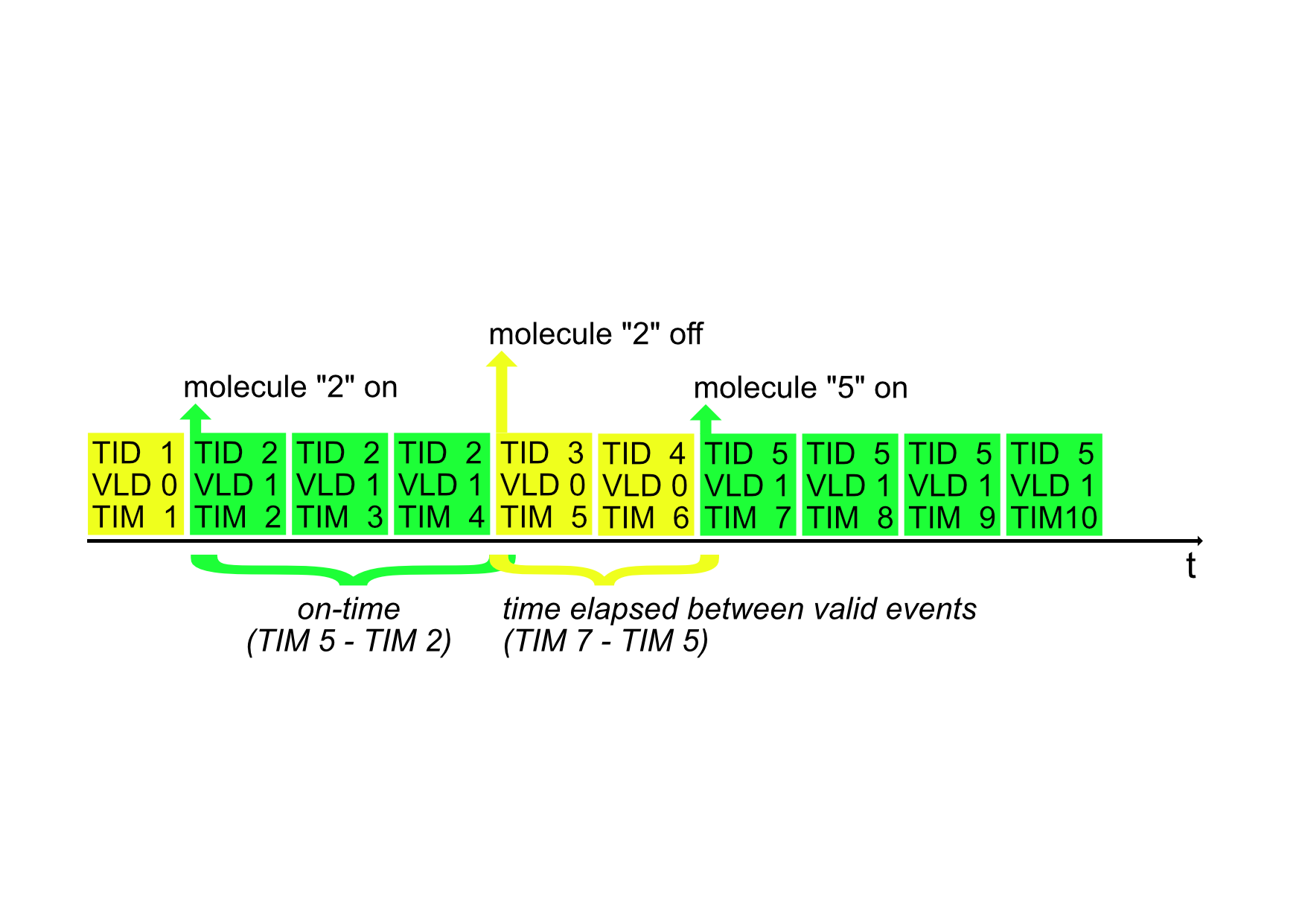


**Time between molecule binding events** $\boldsymbol{t}_{\text{btw}}$ **calculated from the exported measurement parameters.** Saved localization attempts are depicted as colored rectangles, arranged in order of their appearance. Valid localization attempts were saved with the exported parameter VLD = 1 and are shown as green, while the non-valid localization attempts were saved with VLD = 0 and are shown in yellow. The beginning of a localization attempt is saved as a time stamp (exported parameter: TIM), here shown simplified as dimensionless values from 1-10. Localization events belonging to the same molecule have the same trace ID (exported parameter TID). Here, the time between the two consecutive valid molecules is calculated as the time difference between the start of molecule 5 (TIM = 7) and the end of molecule 2 (TIM = 5).

The second quantification parameter was the background emission frequency (*f*_bg_), describing the contribution of unbound imager strands to the localization process. The *f*_bg_ is continuously estimated by the MINFLUX microscope between valid events and is used by the system to identify emission events and to correct emission frequencies of localization events.

The third quantification parameter was the CFR (centre-frequency-ratio). The CFR is the ratio of the effective emission frequency at the central position of the MINFLUX excitation pattern over the mean effective emission frequency over all outer positions and defined as $\mathrm{CFR}=f_{\text{eff}}(\text{central position})/\left\langle f_{\text{eff}}(\text{outer positions}) \right\rangle$. The effective frequencies $f_{\mathrm{eff}}$ are the measured emission frequencies above a background automatically determined by the system. The value of the CFR in the last iteration of localization is regarded as a quality measure for the localization process. For each measurement, the median $\mathrm{CFR}$ of all valid localizations in the last iteration was determined. The $\mathrm{CFR}$ is calculated directly by the microscope software and is also used for filtering in early iterations (exported parameter: CFR). It therefore directly influences the measurement (5).

To estimate the localization precision of a measurement as the third quantification parameter, the standard deviation $\sigma_{r}$ was calculated for each molecule (localizations with the same exported parameter TID) as $\sigma_{r}= \sqrt{\left( \sigma_{x}^{2}+\sigma_{y}^{2} \right)/2}$ with the standard deviations of the $x$- and $y$- coordinates as determined by the microscope (exported parameter POS). The median $\sigma_{r}$ of all molecules with at least 5 localizations was used for the analysis. The combined localization precision was calculated as $\sigma_{rc}=\sigma_{r}/\sqrt{n}$ with $n$ being the number of localization with the same TID.

*CFR simulation.* The CFR is a parameter that is directly calculated during image acquisition by the MINFLUX software. To understand and judge the CFR values from the experimental results we simulated the $\mathrm{CFR}$ dependency on pinhole size and imager concentration for a molecule that is located at the centre of the MINFLUX targeted coordinate pattern (TCP) with background contributions included (see Supplementary notes, Supplementary Note Fig. III). The excitation point spread function (PSF) $h_{\text{exc}}(x,y,z)$ in shape of a 2D donut was determined via fast focus field calculations (6) for high numerical apertures and using realistic values for the objective lens properties as well as an excitation wavelength $\lambda_{\text{exc}}$= 642 nm. The confocal detection PSF $h_{\text{det}}(x,y,z)$ was calculated (7) for a detection wavelength of $\lambda_{\text{exc}}$= 680 nm. We then calculated the resulting effective PSF ${h_{\text{eff},i}=h}_{\text{exc},i}\cdot h_{\text{det}}$ for each exposure $i$ by shifting $h_{\text{exc}}$to the according exposure position in the MINFLUX TCP while keeping the confocal detection $h_{\text{det}}$ centred. The background contribution due to diffusing imager was calculated in two steps. The resulting background intensity $B_{i}$ in the effective excitation volume was calculated as $B_{i}\sim\int_{x,y,z} h_{\mathrm{eff},i}\left( x,y,z \right)\cdot c_{\mathrm{imager}}dxdydz$ for each exposure. For the $\mathrm{CFR}$ calculation we assumed that the central donut exposure of the MINFLUX TCP is placed directly on the molecule, chosen here as the origin. In the case of a perfect donut zero, this leads to a detected emitter intensity of $I_{\mathrm{center}}=0$ for this exposure. The signal intensity detected at different exposures is calculated as $I_{i}\sim h_{\mathrm{eff},i}(0,0,0)$. Correcting for the different total time spent in the inner and outer exposures, the mean background intensity $\overline{B}_{\mathrm{outer}}$and mean signal intensity $\overline{I}_{\mathrm{outer}}$was calculated for the outer exposures ($i\neq1).$ Therefore, we were able to calculate the $\mathrm{CFR}$ as $\mathrm{CFR}= \frac{B_{center}+ I_{center}}{\left\langle\overline{B}_{outer}+\overline{I}_{outer} \right\rangle}$ for different scenarios. We repeated the calculations for different concentrations$c$, adapted the pinhole size when determining $h_{\det}$ and used different values for the TCP diameter $L$.

*Sample drift correction.* Sample drift was corrected from the extracted molecule event position and time pairs by dividing the events into overlapping time windows of approximately 2000 events per window, and generating a 2 or 3D rendered MINFLUX image (placing a Gaussian peak with standard deviation sigma = 2 nm at each estimated molecule position) and calculating 2 or 3D cross-correlations between images from different time windows. The centre of the cross-correlation peak was fitted with a Gaussian function and its offset relative to the centre of the cross-correlation presented the spatial sample shift between the corresponding time points. The drift curve that fulfilled all possible sample drift estimations for all possible time window pairs was estimated in a least squares sense. A smooth (cubic spline) interpolation of the estimated drift curve for all time points of all events was then subtracted from the molecule coordinates.

*FRC calculations.* For the determination of the time evolution of the Fourier ring correlation (FRC) shown in Suppl. Fig. 3 we implemented the algorithm described in (8). In brief, a dataset of combined localizations was divided into two statistically independent subsets resulting in two sub-images, each containing 50 % of the combined localizations of the original data set. Then, the average correlation of the Fourier transform of these sub-images was calculated on rings of constant spatial frequency. The inverse of the spatial frequency at which the FRC drops below 1/7 was taken as a measure of the resolution. We used combined localizations instead of single localizations for the estimation of the FRC resolution, because for single localizations the FRC is dominated by the large number of repeated localizations during one binding event and the calculated FRC resolution is then strictly proportional to the single localization precision.

**Image rendering 2D***.* All valid localization events were rendered using the Imspector Software and displayed as 2D histograms with the bin size 4 nm (Fig. 1 a-f) and 1 nm (Fig. 1 f, close-up).

**Image rendering 3D***.* Each MINFLUX measurement was exported with the Imspector software. The data were drift corrected (see Sample drift correction) and the z position was scaled with the scaling factor 0.7 (9). A rendering of the resulting localizations where each localization was replaced by a Gaussian peak with sigma 5 nm was imported into the Imaris Software (Imaris x64, 9.7.2, Bitplane AG, Zürich, Switzerland). The data were displayed as a blend volume rendition.

**MINFLUX sequences.** The MINFLUX microscope’s data acquisition is controlled by a set of parameters which are specified within a text file (see seqIIF.json and seqDefaultIIF3d.json in the Supplementary data set 1). The set of parameters defines a sequence that controls the iterative zooming in on single molecule events and was provided and optimized by the manufacturer for samples with the dye Alexa Fluor 647. The MINFLUX iteration process is described in detail in (5). In 2D, four iterations plus one pre-localization iteration were performed. In 3D, 9 iterations plus one pre-localization iteration were performed. In the last iteration an *L* of 40 nm was used. Key parameters of the 2D iteration sequence include:

|  | TCP parameter *L* | Photon limit | Dwell time | CFR limit | Laser power factor |
| --- | --- | --- | --- | --- | --- |
| Pre-localization |  | 160 | 1 ms | off | 1 |
| Iteration 1 | 288 nm | 150 | 1-5 ms | 0.5 | 1 |
| Iteration 2 | 151 nm | 100 | 1-5 ms | off | 2 |
| Iteration 3 | 76 nm | 100 | 1-5 ms | 0.8 | 4 |
| Iteration 4 | 40 nm | 150 | 1-5 ms | off | 6 |

**Supplementary Table 1.** Imager concentrations used and localization precisions of the localizations represented in Figure 1 and Figure 2. Localization precisions achievable by combining all localizations of the same event (see: Supplementary Methods/ MINFLUX data analysis/ Quantification).

| Figure | Imager concentration | Localization precision $\sigma_{r}$ $(\sigma_{z})$ | Combined localization precision $\sigma_{rc}$ ($\sigma_{zc})$ |
| --- | --- | --- | --- |
| Figure 1a | 2 nM | 2.7 nm | 0.8 nm |
| Figure 1b | 2.5 nM | 2.6 nm | 0.6 nm |
| Figure 1c | 0.5 nM | 2.3 nm | 0.9 nm |
| Figure 1d | 2 nM | 2.0 nm | 0.6 nm |
| Figure 1e | 2 nM | 2.4 nm | 0.6 nm |
| Figure 1f | 2 nM | 2.7 nm | 0.7 nm |
| Figure 2 TOM70 | 2 nM | 5.5 nm (2.8 nm) | 1.9 nm (0.9 nm) |
| Figure 2 Mic60 | 1 nM | 5.2 nm (3.0 nm) | 1.6 nm (0.9 nm) |
| Figure 2 ATP5B | 1 nM | 5.1 nm (3.0 nm) | 1.5 nm (0.9 nm) |


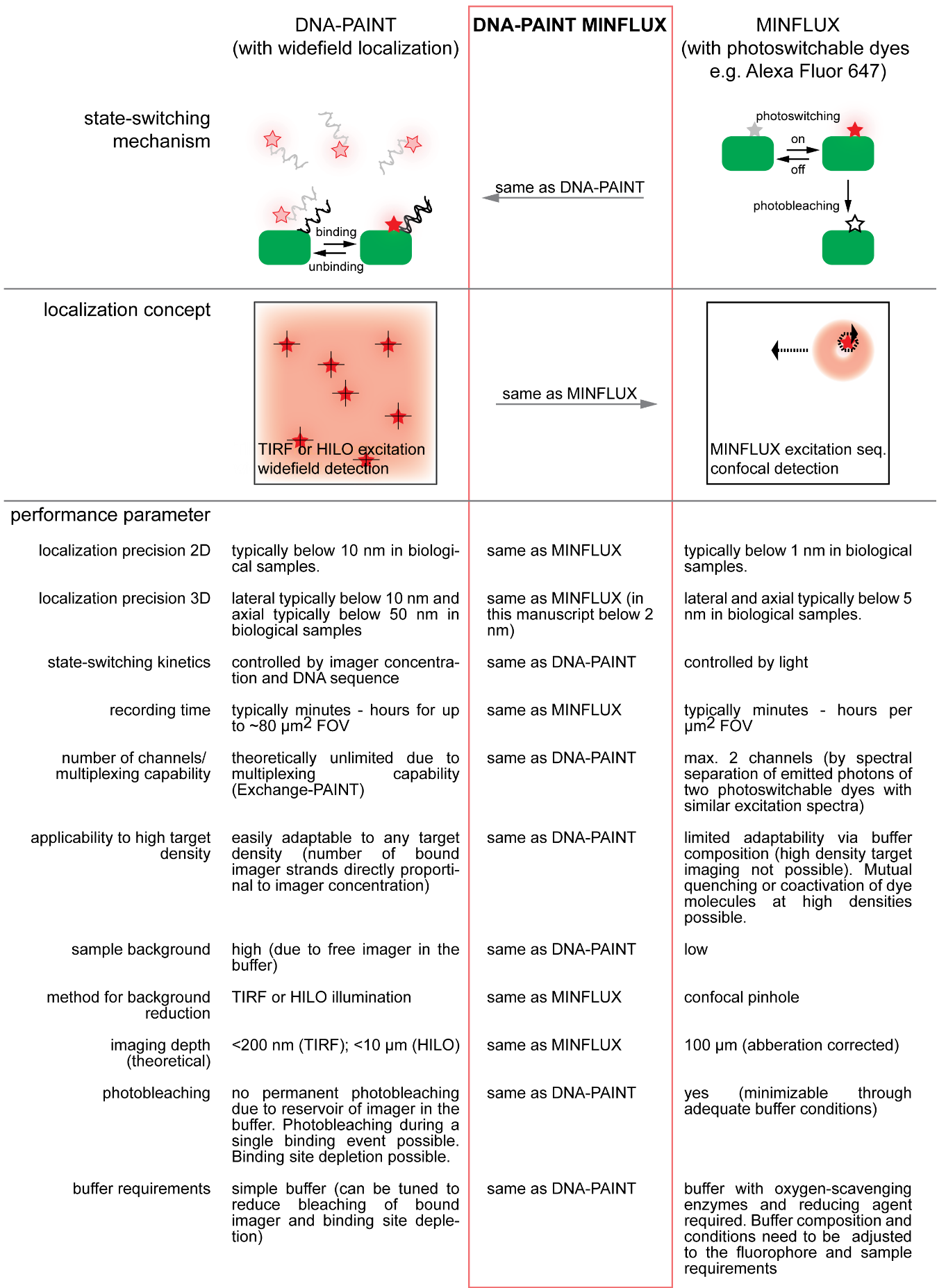


**Supplementary Figure 1. Comparison of current DNA-PAINT, DNA-PAINT MINFLUX and MINFLUX implementations.** The three techniques are compared with respect to their state-switching mechanisms, their localization concepts and key performance parameters.


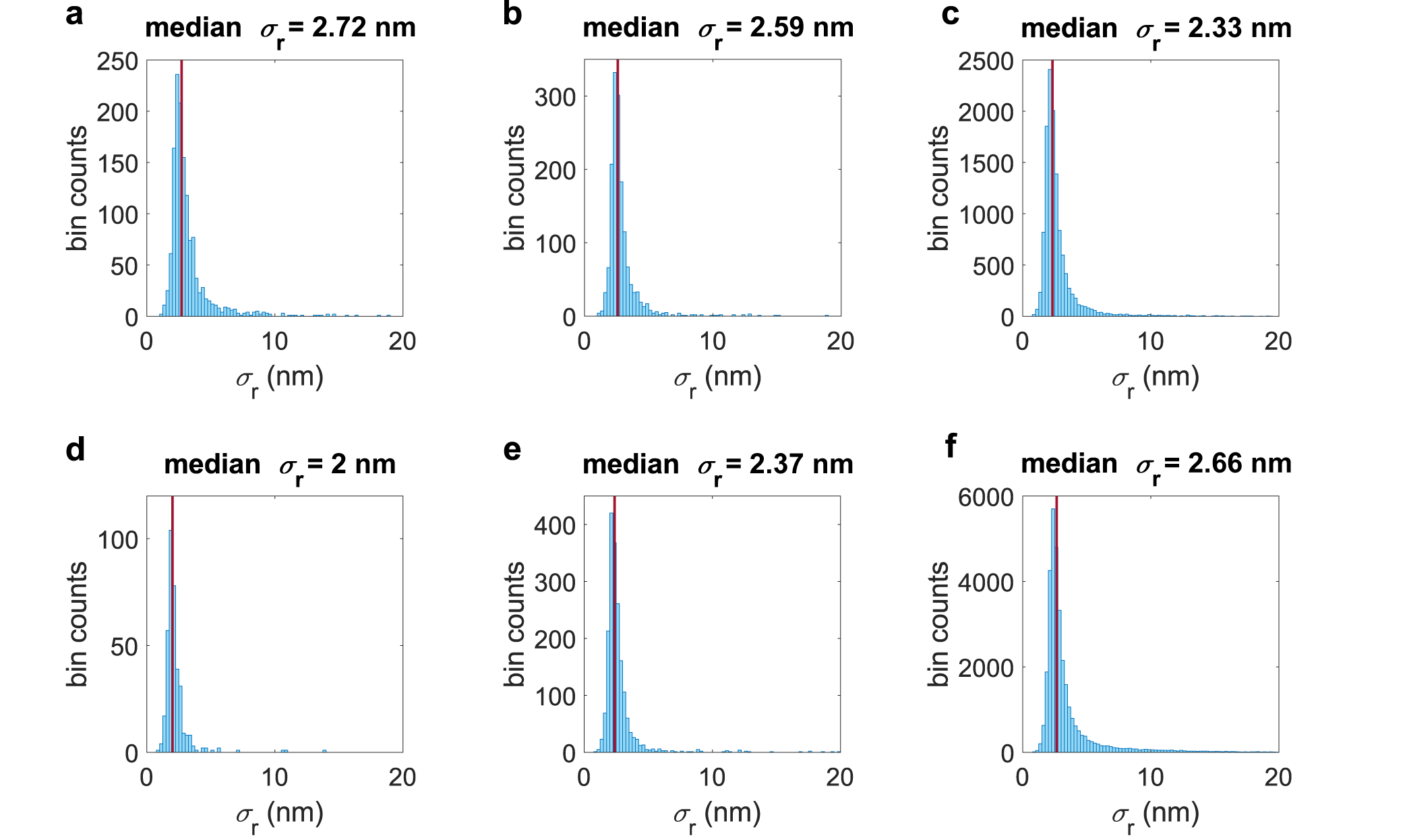


**Supplementary Figure 2. Histograms of localization precisions in Figure 1.** Blue columns represent the frequencies of localization precisions in the given dataset (a: Fig. 1a; b: Fig. 1b; c: Fig. 1c; d: Fig. 1d; e: Fig. 1e; f: Fig. 1f). The red line represents the median of the localization precisions in the dataset.


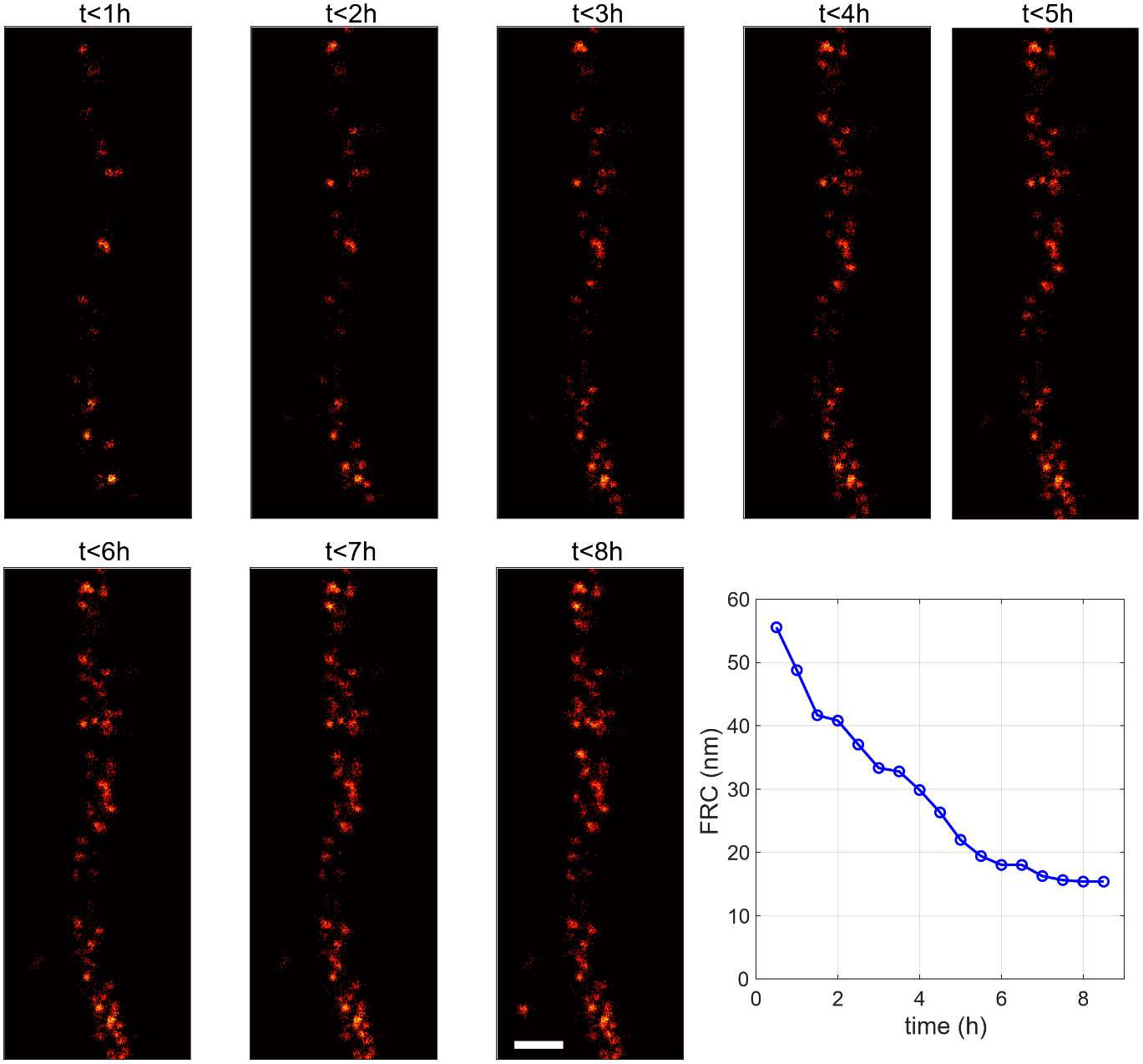


**Supplementary Figure 3. The labelling coverage, but not insufficient sampling during a MINFLUX recording, limits the density of localized molecules.** The individual panels show all recorded localizations in the indicated time intervals. The 8-hour data set is shown in Fig. 1f. The FRC resolution was calculated using all data up to a certain time point (blue circles). After 6-7 h almost no new localizations contribute to the recorded Vimentin filament and the FRC resolution reaches a plateau, suggesting that the imaging time was sufficient to localize the vast majority of available binding sites. Scale bar: 50 nm.

# **Supplementary notes**

## **Performance indicators of DNA-PAINT MINFLUX recordings**

We systematically explored the influence of the experimental key variables (laser power, pinhole size and imager concentration) on DNA-PAINT MINFLUX recordings. Specifically, the influence on the time between valid events (*t*_btw_), the background emission frequency (*f*_bg_), the center-frequency-ratio (CFR) and the localization precision (*σ_r_*) were determined, as together these four parameters provide a measure of the image quality, the average success of the localization processes, and the time for recording a MINFLUX image. These parameters are calculated according to their definition given in Supplementary Methods, MINFLUX 2D Analysis.

The idle time between two valid molecule binding events (*t*_btw_) is a major determinant of the overall recording speed in MINFLUX nanoscopy, as the molecules are recorded sequentially.

The background emission frequency (*f*_bg_) is continuously estimated by the MINFLUX microscope in between valid events and is used by the system to identify emission events and to correct emission frequencies of localization events.

The center-frequency-ratio (CFR*)* is a parameter calculated during image acquisition by the MINFLUX software and is used as an internal abort criterion in the first and the third MINFLUX iteration steps at each localization attempt. The CFR is defined as the ratio between the effective, background corrected emission frequency determined at the central position of the MINFLUX excitation donut over the average effective emission frequency at the outer positions. The CFR is small when the central position of the probing donut is placed on the molecule and the CFR increases when the central position of the donut in the MINFLUX targeted coordinate pattern (TCP) deviates from the molecule position. Its value is also influenced by the effectiveness of the background correction.

Because the CFR is only a general indicator for the localization quality, we also directly determined the localization precision in the measurements. To estimate the average localization precision within one measurement, we choose the median of $\sigma_{r}$ of all events.

To systematically characterize the influence of varying excitation laser powers, pinhole sizes and imager concentrations on *t*_btw_, *f*_bg_, CFR and *σ_r_* we recorded DNA-PAINT MINFLUX images of a well-established cellular intracellular model structure, namely nuclear pores in cultivated human cells. To this end, genome edited HeLa cells expressing mEGFP-Nup107 were chemically fixed and labeled with anti-GFP nanobodies that were coupled to a docking DNA-oligo. The complementary DNA-oligo coupled to Atto655 was used as an imager. Within one quantification measurement series (see also Supplementary Methods, MINFLUX measurements), all experimental variables but one were kept constant. All measurements within a series were repeated three times on different days.

## **Influence of the laser power on MINFLUX performance**


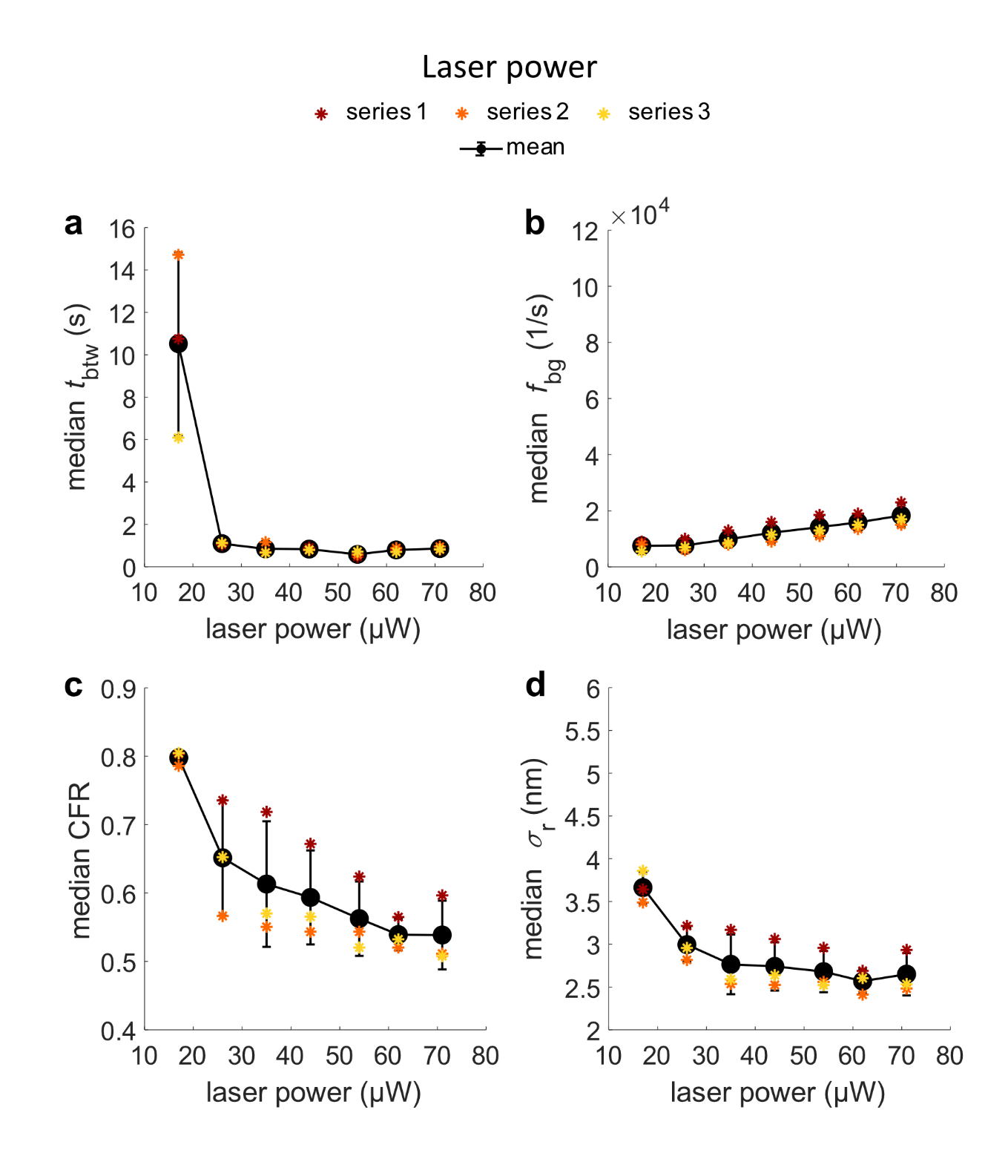
**Supplementary Note Figure I. Influence of the laser power on the parameters *t*_btw_ , *f*_bg_, CFR and** $\boldsymbol{\sigma}_{\boldsymbol{r}}$**.** Nup107-mEGFP cells were fixed and labelled with an anti-GFP nanobody coupled to a docking strand. 1 µm^2^ ROIs were imaged for an hour each, using a pinhole size of 0.45 AU and an imager concentration of 2 nM. The laser powers given refer to the power in the sample at the first iteration of the MINFLUX sequence. At the last iteration of the sequence, the power is six times higher. Colored asterisks represent the median of the respective parameter within one measurement series. Black dots represent the mean of the three measurement series and error bars represent the standard deviation from the mean.

In the MINFLUX sequence used, the laser power is increased six fold from the first to the final iteration. Consequently, the initial laser power could be maximally set to 71 µW (in the sample; 16% of the available laser power). To characterize the influence of the laser power on *t*_btw_, *f*_bg_, CFR and *σ_r_* we varied the laser power between 17 and 71 µW in the first iteration (4 % - 16 %), which also corresponds to a variation of the laser power in all other iterations. In this measurement series, all images were taken with an imager concentration of 2 nM and a pinhole size of 0.45 Airy units (AU).

At laser powers below 26 µW in the first iteration (6 %), the $t_{btw}$ increased, presumably, because at too low laser intensities the likelihood of events with sufficient detected photons to cross the photon thresholds in the MINFLUX iteration scheme decreases (Supplementary Note Fig. Ia). Above a minimal threshold, the *t*_btw_ was largely independent of the laser power. This can be attributed to the fact that in DNA-PAINT the single-molecule event kinetics are primarily determined by the binding kinetics of the imager to the docking strand, and not by activation light, as in previous MINFLUX implementations.

Higher laser powers led to an increased *f*_bg_ (Supplementary Note Fig. I b). This can be explained by a stronger excitation of free imager in the sample.

With increasing laser power, the experimentally observed median $\mathrm{CFR}$ decreased (Supplementary Note Fig. I c). For a background-free DNA-PAINT MINFLUX measurement, we would expect the CFR to be independent of the laser power. However, when imaging a real biological sample, background is inevitable. In DNA-PAINT background is especially high due to the free imager in the sample. The MINFLUX software applies an automated adaptive background correction on the estimation of the CFR. As we observe a decrease of the CFR with increasing laser power, we assume that the algorithm does not completely correct for the background.

Similar to the $\mathrm{CFR}$, also the median of *σ_r_* slightly decreased with increasing laser power (Supplementary Note Fig. I d). This is likely a side effect of the finite dwell time per targeted coordinate, which at higher laser powers results in a slightly higher number of collected photons above the threshold that must be reached for the localization to be accepted.

## **Influence of the detection pinhole size on MINFLUX performance**


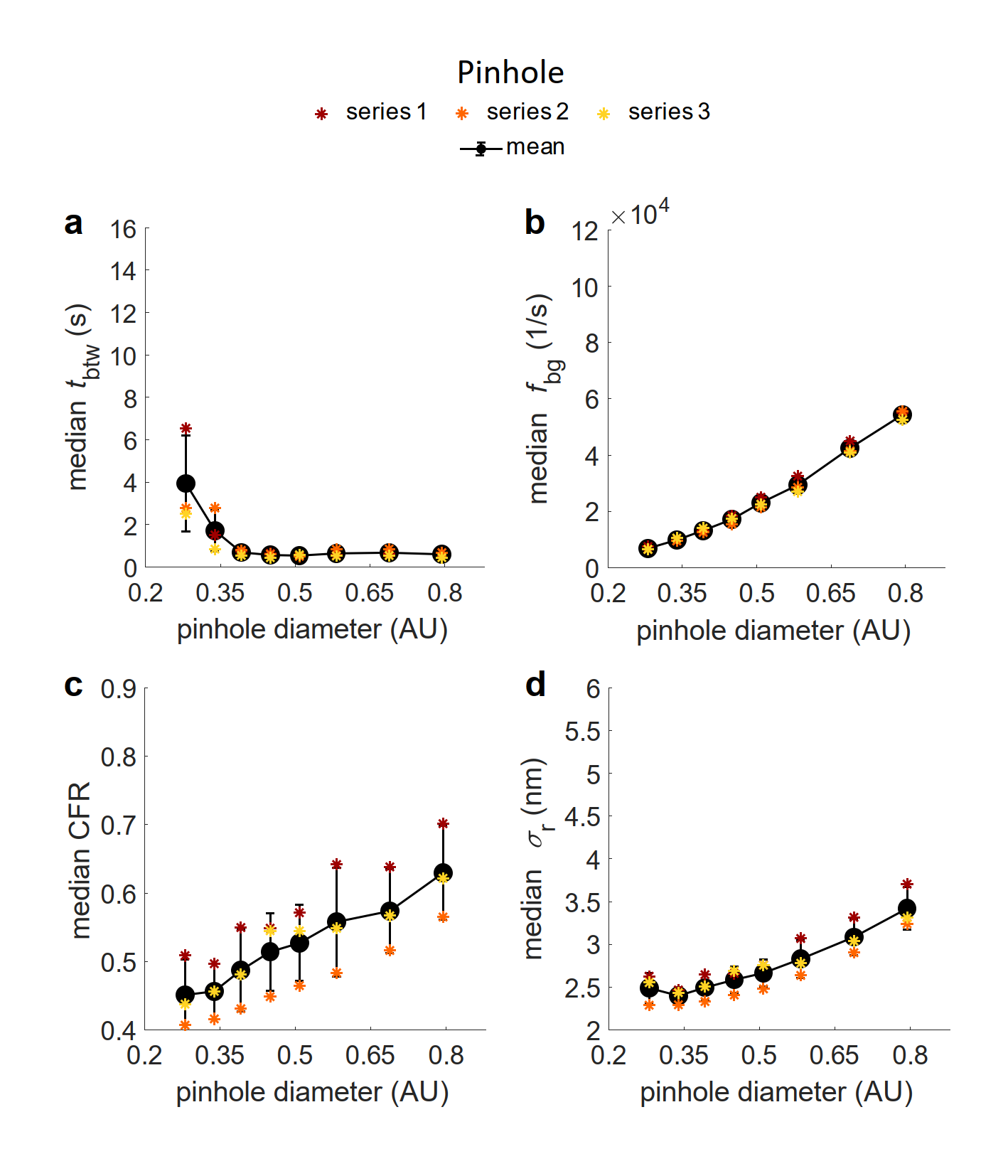


**Supplementary Note Figure II. Influence of the pinhole size on the parameters *t*_btw_, *f*_bg_, CFR and *σ_r._*** Nup107-mEGFP cells were fixed and labelled with an anti-GFP nanobody coupled to a docking strand. 1 µm^2^ ROIs were imaged for an hour each, using a laser power of 71 µW in the sample in the first iteration and an imager concentration of 2 nM. Colored asterisks represent the median of the respective parameter within one measurement series. AU: Airy units. Black dots represent the mean of the three measurement series and error bars represent the standard deviation from the mean.

We analysed the influence of different pinhole sizes on *t*_btw_, *f*_bg_, CFR and *σ_r._*. For this, we chose to vary the size of the pinhole in a range of 0.28 - 0.79 AU. The images were recorded with a laser power of 71 µW in the first iteration and 2 nM imager concentration.

Above a threshold (~ 0.4 AU), we found *t*_btw_ to reach a constant plateau (Supplementary Note Fig. II a). The increase of *t*_btw_ at small pinhole sizes is expected, as when decreasing the pinhole size, not only background photons are rejected, but also photons emitted by the localized molecule. Consequently, less and less signal is detected until an increasing number of localization attempts no longer passes the photon thresholds of the MINFLUX iteration sequence.

The *f*_bg_ increased with larger pinhole sizes (Supplementary Note Fig. II b). This is immediately explained by increased photon counts from the free imager in the buffer

The $\mathrm{CFR}$ increased almost linearly with the pinhole size (Supplementary Note Fig. II c). Calculations that take into account an increasing background related to the pinhole size but do not consider an adaptive background correction, also suggest an approximately linear relationship between pinhole size and CFR (Supplementary Note Fig. III), similar to the measured data. Again, this observation suggests that the background subtraction performed by the MINFLUX software does not fully compensate for the background when using DNA-PAINT. With smaller pinhole sizes, the experimentally determined localization precision improved (Supplementary Note Fig. II d) down to a pinhole size of 0.34 AU. At even smaller pinhole sizes, presumably too few photons were detected to improve $\sigma_{r}$ further.


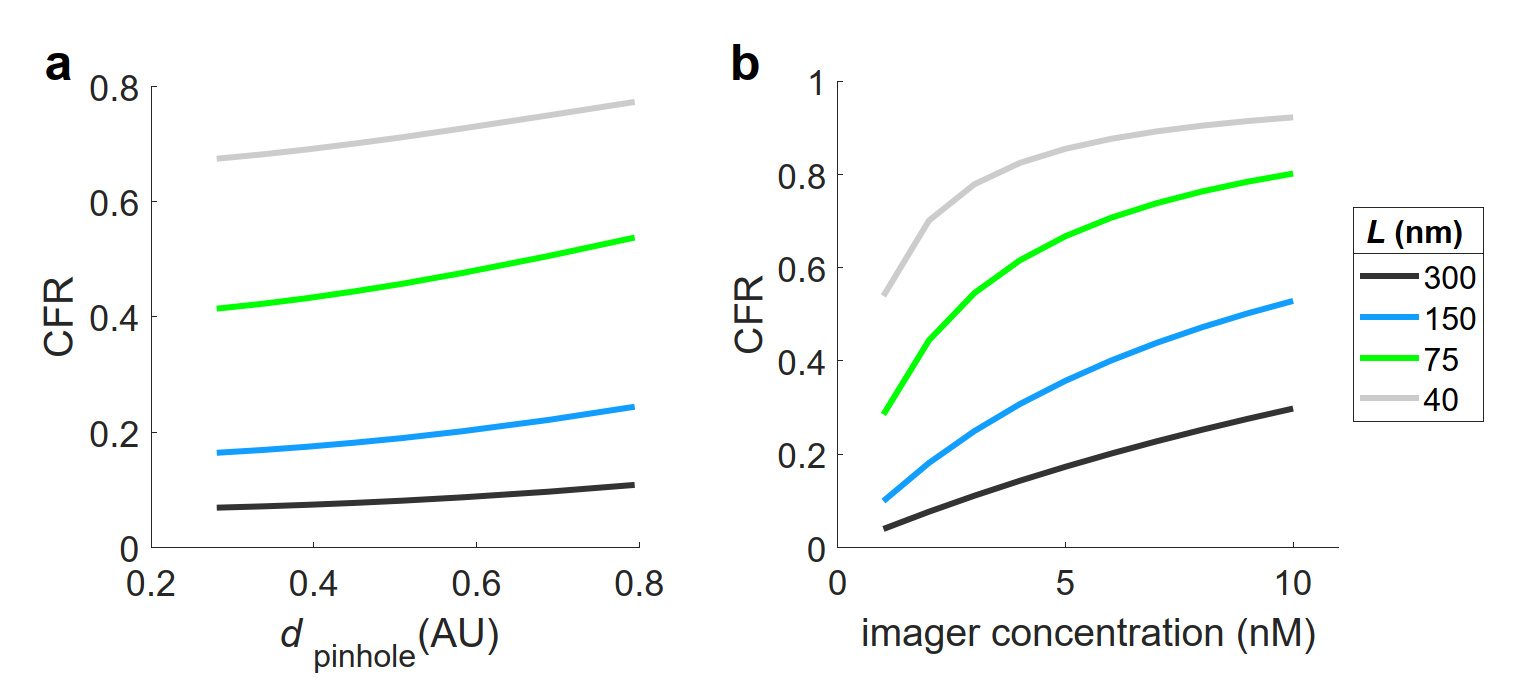


**Supplementary Note Figure III. CFR simulations for varying pinhole diameter at 2 nM imager concentration (a) and varying imager concentration at a pinhole size of 0.45 AU (b).** The CFR was calculated in both cases as described in (Supplementary Methods, CFR Calculations) for one MINFLUX iteration using a targeted coordinate pattern (TCP) with one central exposure and six outer exposures arranged on a circle with diameter L.

## **Influence of imager concentration on MINFLUX performance**


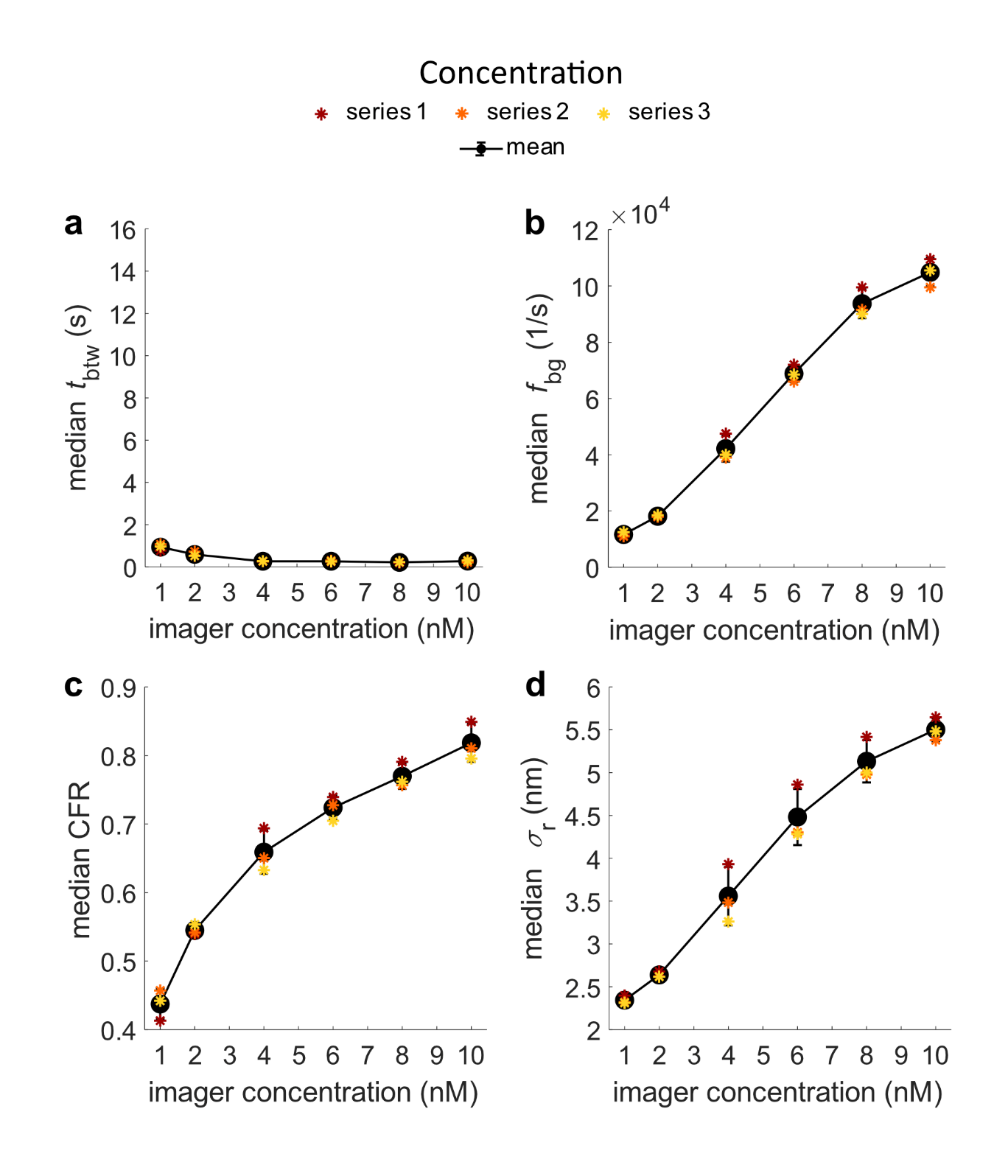


**Supplementary Note Figure IV. Influence of the imager concentration on the parameters *t*_btw_, *f*_bg_, CFR and** $\boldsymbol{\sigma}_{\boldsymbol{r}}$**.** Nup107-mEGFP cells were fixed and labelled with an anti-GFP nanobody coupled to a docking strand. 1 µm^2^ ROIs were imaged for an hour each, using a pinhole size of 0.45 AU and a laser power of 71 µW in the sample in the first iteration. Colored asterisks represent the median of the respective parameter within one measurement series. Black dots represent the mean of the three measurement series and error bars represent the standard deviation from the mean.

The influence of the imager concentration on the DNA-PAINT MINFLUX imaging parameters was analysed. The imager strand concentration was varied between 1 and 10 nM. The laser power was set to 71 µW in the first iteration, and a pinhole size of 0.45 AU was used.

At very low imager concentrations, *t*_btw_ increased (Supplementary Note Fig. IV a). This was expected, as the number of binding events scales linearly with the concentration of the imager at low concentrations. Above ~ 4 nM imager, *t*_btw_ reached a plateau. This demonstrates that *t*_btw_ is rather insensitive against the imager concentration, once the lower threshold is passed. We predict that the *t*_btw_ might increase again at higher imager concentrations outside of the tested concentration range, because we expect at very high imager concentrations an increasing number of aborted localization events due to multiple fluorophores binding within the examined MINFLUX localization region.

The *f*_bg_ increased with higher imager concentrations (Supplementary Note Fig. IV b). A linear dependence of background on imager concentration is to be expected. However, the shape of the curve indicates a slightly non-linear relationship, suggesting a not fully functional background detection by the microscope software in DNA-PAINT.

The CFR increased with increasing imager concentrations (Supplementary Note Fig. IV c). Computing this relationship without background correction, assuming a background intensity $I_{bg}(c)$ which depends linearly on the imager strand concentration and a background independent molecule intensity $I_{m}$, results in $\mathrm{CFR}\left( c \right)\sim\frac{I_{bg} (c)}{I_{bg} \left( c \right)+I_{m}}$, which reflects the experimental data well for small diameter $L$ of the MINFLUX excitation pattern (Supplementary Note Fig. III).

The localization precision decreases with an increasing imager concentration (Supplementary Note Fig. IV d). We assume that with higher imager concentrations not only the background increases, but also the likelihood of a second imager molecule binding in spatial proximity to a localized binding event rises. These two factors will result in the decrease of the median *σ_r._*

## **Optimal parameter selection in DNA-PAINT MINFLUX**

Together, these data show that in DNA-PAINT MINFLUX imaging an appropriate imager concentration is a key determinant of the localization precision. However, at too low imager concentrations *t*_btw_ increases strongly. The pinhole size has opposed effects on the localization precision and on *t*_btw_, requiring the identification of an optimal pinhole size. The localization precision scales inversely with increasing laser power, and at the laser intensities available, we did not observe any decrease of *t*_btw_ above a threshold of 26 µW in the first iteration. The MINFLUX microscope largely behaves as expected for an imaging system with only partial background subtraction in the estimation of the CFR.

In conclusion, a good starting point for DNA-PAINT MINFLUX measurements using Atto655 is a laser power of at least 62 µW in the first iteration, a pinhole size of 0.45 AU and, for nuclear pore imaging, an imager concentration of 2 nM (the imager concentration has to be adapted to the target binding sites density).

For the use of other dyes, the imaging parameters presumably need to be adjusted. This study shows that the imager background is a major factor influencing the localization performance in DNA-PAINT MINFLUX nanoscopy. Therefore, it is advisable to start optimizing parameters with a low imager concentration (without extending the recording time to unacceptable values). A small pinhole should be chosen, and a sufficiently high laser power is required to collect enough photons during one binding event.

## **Possible further improvements**

DNA-PAINT MINFLUX nanoscopy has distinct advantages over conventional MINFLUX nanoscopy, most notably the possibility of unlimited multiplexing and the lack of a need for dedicated buffer adjustments.

However, free imager causes an increase in the background emission frequency (*f*_bg_), and the challenge of long recoding times remain. Both challenges are also known from standard DNA-PAINT nanoscopy.

Several approaches to reduce the background problem in DNA-PAINT nanoscopy have been reported. This includes the use of Förster resonance energy transfer (FRET)-based probes (10, 11), caged, photactivatable dyes (12), as well as fluorogenic DNA-PAINT probes (13). Presumably, these or related approaches would also benefit DNA-PAINT MINFLUX nanoscopy.

In the DNA-PAINT implementation used in this study, we relied on standard, commercially available imager strands. Thereby we localized on average each molecule more than 20 times, while the imager strand was bound to the docking strand. That many localizations have only limited benefit to the average localization precision, but are time consuming. Hence, a probe with a moderately higher off-rate would presumably save time without unacceptably deteriorating the localization precision.

Using a probe with a higher on-rate would additionally allow for lower imager concentration and thereby reduce the background, without extending the idle time between two valid molecule binding events (*t*_btw_). Indeed, several studies report on the design of optimized DNA sequences and buffer optimization in order to minimize the time between events in DNA-PAINT nanoscopy and thereby accelerate the recording time (14-17). This resulted in an up to 100-fold speed-up in imaging (16). Other concepts to accelerate DNA-PAINT nanoscopy relied on the preloading of DNA-PAINT imager strands with Argonaute proteins (18).

In addition to accelerating the recoding time by modulating the binding kinetics of the imager strand, we assume that there is potential in tailoring the MINFLUX sequence to DNA-PAINT labelling. For this study, we relied on the generic MINFLUX sequence provided by the microscope manufacturer. This has not been optimized for DNA-PAINT and we assume that substantial improvements in imaging time and localization quality are possible when this sequence would be specifically tailored. Concretely, the number of iterations, the photon thresholds, the number of localization attempts for one event and the sizes of the TCP diameter *L* in the iterations could be adapted.

Ultimately, we predict that accelerating MINFLUX nanoscopy will require parallelization of the localization process by changing the instrument design.

# **References**

1. M. Ratz, I. Testa, S. W. Hell, S. Jakobs, CRISPR/Cas9-mediated endogenous protein tagging for RESOLFT super-resolution microscopy of living human cells. *Sci Rep* **5**, 9592 (2015).

2. J. V. Thevathasan *et al.*, Nuclear pores as versatile reference standards for quantitative superresolution microscopy. *Nat Methods* **16**, 1045-1053 (2019).

3. S. Otsuka *et al.*, Nuclear pore assembly proceeds by an inside-out extrusion of the nuclear envelope. *Elife* **5** (2016).

4. F. Balzarotti *et al.*, Nanometer resolution imaging and tracking of fluorescent molecules with minimal photon fluxes. *Science* **355**, 606-612 (2017).

5. R. Schmidt *et al.*, MINFLUX nanometer-scale 3D imaging and microsecond-range tracking on a common fluorescence microscope. *Nat Commun* **12**, 1478 (2021).

6. M. Leutenegger, R. Rao, R. A. Leitgeb, T. Lasser, Fast focus field calculations. *Opt Express* **14**, 11277-11291 (2006).

7. M. Leutenegger, T. Lasser, Detection efficiency in total internal reflection fluorescence microscopy. *Opt Express* **16**, 8519-8531 (2008).

8. R. P. Nieuwenhuizen *et al.*, Measuring image resolution in optical nanoscopy. *Nat Methods* **10**, 557-562 (2013).

9. K. C. Gwosch *et al.*, MINFLUX nanoscopy delivers 3D multicolor nanometer resolution in cells. *Nat Methods* **17**, 217-224 (2020).

10. A. Auer, M. T. Strauss, T. Schlichthaerle, R. Jungmann, Fast, Background-Free DNA-PAINT Imaging Using FRET-Based Probes. *Nano Lett* **17**, 6428-6434 (2017).

11. J. Lee, S. Park, W. Kang, S. Hohng, Accelerated super-resolution imaging with FRET-PAINT. *Mol Brain* **10**, 63 (2017).

12. S. Jang, M. Kim, S. H. Shim, Reductively Caged, Photoactivatable DNA-PAINT for High-Throughput Super-resolution Microscopy. *Angew Chem Int Ed Engl* **59**, 11758-11762 (2020).

13. K. K. Chung *et al.*, Fluorogenic probe for fast 3D whole-cell DNA-PAINT. *bioRxiv* 10.1101/2020.04.29.066886, 2020.2004.2029.066886 (2020).

14. M. Schickinger, M. Zacharias, H. Dietz, Tethered multifluorophore motion reveals equilibrium transition kinetics of single DNA double helices. *Proc Natl Acad Sci U S A* **115**, E7512-E7521 (2018).

15. F. Schueder *et al.*, An order of magnitude faster DNA-PAINT imaging by optimized sequence design and buffer conditions. *Nat Methods* **16**, 1101-1104 (2019).

16. S. Strauss, R. Jungmann, Up to 100-fold speed-up and multiplexing in optimized DNA-PAINT. *Nat Methods* **17**, 789-791 (2020).

17. A. H. Clowsley *et al.*, Repeat DNA-PAINT suppresses background and non-specific signals in optical nanoscopy. *Nat Commun* **12**, 501 (2021).

18. M. Filius *et al.*, High-Speed Super-Resolution Imaging Using Protein-Assisted DNA-PAINT. *Nano Lett* **20**, 2264-2270 (2020).
